# Supplementary material for: Individualised Exercise Training Enhances Antioxidant Buffering Capacity in Idiopathic Pulmonary Fibrosis
Source: Antioxidants (Basel). 2023 Aug 20;12(8):1645. doi: 10.3390/antiox12081645 (PMC10451244; doi:10.3390/antiox12081645)
Supplement: Supplementary file 1 [file antioxidants-12-01645-s001.zip › antioxidants-2562429-supplementary.pdf]

Supplementary Materials

Individualised Exercise Training Enhances Antioxidant Buffering Capacity in Idiopathic Pulmonary Fibrosis

Tim J. M. Wallis [1,2,*], Magdalena Minnion [1,2], Anna Freeman [1,2], Andrew Bates [1,2,3], James M. Otto [1,2,3], Stephen A. Wootton [2,4], Sophie V. Fletcher [1,2], Michael P. W. Grocott [1,2,3], Martin Feelisch [1,2,†], Mark G. Jones [1,2,5,†] and Sandy Jack [1,2,3,†]

[1] NIHR Southampton Biomedical Research Centre, Respiratory and Critical Care, University Hospital Southampton, Southampton SO16 6YD, UK; m.minnion@soton.ac.uk (M.M.); a.freeman@soton.ac.uk (A.F.); a.bates@soton.ac.uk (A.B.); james.otto@uhs.nhs.uk (J.O.); sophie.fletcher@uhs.nhs.uk (S.V.F.); mike.grocott@soton.ac.uk (M.P.W.G.); m.feelisch@soton.ac.uk (M.F.); mark.jones@soton.ac.uk (M.G.J.); s.jack@soton.ac.uk (S.J.)

[2] Academic School of Clinical and Experimental Sciences, Faculty of Medicine, University of Southampton, Southampton SO17 1BJ, UK; s.a.wootton@soton.ac.uk

[3] Department of Critical Care and Anaesthesia, University Hospital Southampton, Southampton SO16 6YD, UK

[4] NIHR Southampton Biomedical Research Centre, Nutrition and Metabolism, University Hospital Southampton, Southampton SO16 6YD, UK

[5] Institute for Life Sciences, University of Southampton, Southampton SO17 1BJ, UK

* Correspondence: tim.wallis@uhs.nhs.uk

† These authors contributed equally to this work.

Supplemental File Contents

1: Clinical outcome protocols 3

1.1 Incremental Cardiopulmonary Exercise test (CPET) 3

1.2 Constant work-rate exercise test (CWRT) 3

1.3 Spirometry and Gas transfer assessment 3

1.4 Medical Research breathlessness scale 3

1.5 St. George's respiratory questionnaire 3

2: Structured Responsive Exercise Training Programme 4

2.1 Description of the SRETP following the Consensus on Exercise Reporting Template (9) 4

2.2 Tabulated representation of the SRETP 6

2.3 Visual summary of the SRETP 6

3: Biomarker analytical methodology 7

4: Expressing the oxygen cost of exercise 9

Supplementary Tables 10

Supplementary Table S1 10

Supplementary Table S2 10

Supplementary Table S3 11

Supplementary Table S4 12

Supplementary Table S5 13

Supplementary Table S6 13

Supplementary Table S7 14

Supplementary Table S8 15

Supplementary Table S9 16

Supplementary Table S10 16

Supplementary Figures 17

Supplementary Figure S1 17

Supplementary Figure S2 18

Supplementary Figure S3 19

Supplementary References 20

1: Clinical outcome protocols

1.1 Incremental Cardiopulmonary Exercise test (CPET)

Incremental cardiopulmonary exercise test (CPET) followed protocol and guidelines of the perioperative exercise testing and training society (POETs) (1) and the American Thoracic Society/American College of Chest Physicians.(2) Identical protocol was employed for both IPF and Control participants. Safety criteria for the test were employed as documented in these guidelines. For each patient the estimated ramp slope was first calculated using established formula derived by Wasserman et al. (3) then adjusted pragmatically based on the patients physiological status to obtain a test time of approximately 8-12 minutes. During incremental CPET active encouragement was given to patients to facilitate best individual performance in the test. The test was terminated either by the patient indicating that they had reached their symptom limitation or if their pedalling cadence fell to less than 50 rpm for greater than 10 s. For subsequent CPETs the same ramp slope as baseline was employed. $\dot{V}O_2$peak percent predicted values were calculated from equations derived by Hansen and Wasserman.(3) All CPETs were conducted in the morning or early afternoon (0800-1300) and patients were instructed to attend the research centre fasted and provided with a light breakfast of toast, butter, spread, jam or cheese at least 2 hours prior to the test. This dietary restriction was necessary to control for nitrogen containing foods which would influence blood analysis of redox markers. One participant was taking the antioxidant N-acetyl cysteine (NAC) at a dose of 600 mg three times a day.

1.2 Constant work-rate exercise test (CWRT)

The CWRT was performed at a separate visit at least 48 hours after incremental CPET (**Supplemental Figure 1**). The CWRT was performed with near identical protocol to CPET. However instead of the incremental ramp phase following unloaded pedalling the work-rate of the test (75% of work rate achieved at $\dot{V}O_2$peak during baseline incremental CPET) was introduced 'on-block'. After baseline CWRT patients were not informed of their achieved Endurance time to help prevent bias at the final assessment of 'having a target' to achieve. The test was terminated either by the patient indicating that they had reached their symptom limitation or if pedalling cadence fell to less than 50 rpm for greater than 10 s.

1.3 Spirometry and Gas transfer assessment

All spirometry was reported to reference ranges of the Global Lung Initiative (GLI) 2012(4) and technical specifications of the European Respiratory Society (ERS) and ATS.(5) Single-breath carbon monoxide uptake in the lung testing is reported to technical standards of the ERS and ATS 2017(6).

1.4 Medical Research breathlessness scale

The Medical Research Council breathlessness score was completed as a patient reported outcome of symptomatic breathlessness. The Medical Research Council Breathlessness score adapted from (7):

Grade	Degree of breathlessness related to activities
1	Not troubled by breathlessness except on strenuous exercise
2	Short of breath when hurrying on the level or walking up a slight hill
3	Walks slower than most people on the level, stops after a mile or so, or stops after 15 minutes walking at own pace
4	Stops for breath after walking about 100 yards, or after a few minutes on level ground
5	Too breathless to leave the house, or breathless when undressing

1.5 St. George's respiratory questionnaire

The IPF Specific version of the St. Georges respiratory questionnaire was used as a validated patient reported health related quality of life assessment tool.(8)

2: Structured Responsive Exercise Training Programme

2.1 Description of the SRETP following the Consensus on Exercise Reporting Template (9)

Type of exercise equipment used

Exercise training consisted of 30 min (Week 1) and 40 min (Week 2-8) -including 5 min warm-up and 5 min cool-down- of interval training on an electromagnetically braked cycle ergometer (Optibike Ergoline GmbH, Germany). The training programme was preloaded on a chip-and-pin card which executed the interval intensities automatically.

Qualifications, teaching/supervising expertise, and/or training undertaken by the exercise instructor

The exercise intervention was supervised by hospital staff with a minimal qualification of Basic Life Support. These included physiotherapists, sports medicine practitioners, research nurses and study doctors. Each participant's first exercise session was supervised by a medically trained clinician. A named medically trained clinician was available on-site during training sessions.

Describe whether the exercises are performed individually or in a group

Wherever possible, dependent on availability, trial participants were exercised in pairs, to provide camaraderie.

Exercise supervision and delivery

Prior to each exercise session participants were asked if they felt well enough to perform the exercise session, asked about possible intercurrent illness, and had focused physical examination and vital signs recorded. During the session the participants were reminded to inform the supervisor of chest pain, sudden shortness of breath and dizziness as the POETs guidelines. During exercise training session continuous heart rate was monitored by R-R interval and peripheral oxygen saturation recorded. The study protocol stated that if oxygen saturations dropped to <85% during sessions participants were required to rest until recovery to baseline levels however no participants met this threshold during training sessions.

How fidelity/ adherence to exercise was measured and reported

Adherence was reported as the number of exercise sessions completed divided by the total number of sessions available (n=16). In the study protocol a minimum of 12 out of 16 sessions (≥75%) was pre-specified as the minimum adherence required to complete the programme.

Description of motivation strategies

All exercise sessions were supervised throughout as described above. No specific motivational strategies were adopted although participants were given gentle positive encouragement through the exercise sessions.

Exercise progression

Exercise intensity is prescribed onto a chip and pin card, using the participant's physiological variables determined by CPET. To ensure that progressive overload was maintained throughout the trial, participants underwent CPET after 4 weeks and the training programme was modified for each individual's ramped CPET protocol results ensuring consistent, progressive and individualized intensities for all subjects.

Detailed description of the exercise training sessions

Exercise training consisted of 30 min (Week 1) and 40 min (Week 2-8) -including 5 min warm-up and 5 min cool-down- of interval, aerobic training on an electromagnetically braked cycle ergometer (Optibike Ergoline GmbH, Germany). All IPF participants were invited to attend an outpatient based, in-hospital, 8 week, twice weekly structured, responsive exercise training programme. Participants wore a heartrate monitor throughout the session which reported pulse rate onto the ergometer screen. Training intensities were prescribed onto a chip and pin card, based on individual's physiological variables, (anaerobic threshold (AT) and $\dot{V}O_2$peak) determined at CPET. This card was inserted into the cycle ergometer and participants instructed to pedal at a cadence of 60-65 revolution per minute, according to a visible reading. Sessions lasted for 30 min (Week 1) or 40 min (Week 2-8), starting with a 5-minute warm-up at 0-5 watts. After 5 minutes warm-up, the interval components began with 3 minutes, moderate intensity, at a work rate equal to 80% of that achieved at anaerobic threshold (AT). This was followed by high intensity exercise, for 2 minutes at a work rate equal to 50% of the difference in work rate between AT and $\dot{V}O_2$peak (for graphical representation of training session see **Supplementary Section S2.2**). This 5-minute interval was then repeated; 4 times (Week 1) or 6 times (Week 2-8), followed by a 5-minute recovery period at 0-5 watts. In total, exercise sessions lasted for 30-40 minutes as described. Progress through the session, pedalling cadence, and heart rate were visible to the participant on a digital display at the head of the cycle ergometer (**see Supplementary Section 2.3**).

Home programme/non-exercise component

All study exercise participation took place in-hospital as an out-patient. No home exercises were prescribed, and participants were instructed to continue with only their usual daily activities in addition to the exercise programme for the duration of the study. No non-exercise components were included in this study.

Type and number of adverse events

Exercise related adverse events would be deemed attributable if occurring within 30 minutes of exercise or CPET. No serious adverse events were reported. 2 mild (Grade 1) adverse events were reported; 1 after CPET and 1 after an exercise training session. Both mild post-exercise presyncope which resolved after oral hydration and a brief period of observation.

Setting in which the exercise was performed

Exercise sessions were performed in an outpatient, in-hospital setting, within a designated research physiology laboratory.

Extent of adherence

During the study the median adherence of the 11 participants who completed the SRETP was 88% (min. 75% - max. 94%). All 11 participants achieved ≥75% adherence. 2 participants were within the exercise training programme at point of study termination due to the COVID-19 pandemic. The individual adherence for each of these individuals (factored for the number of weeks training completed) was 75% (9/12 sessions) and 100% (4/4 sessions).

2.2 Tabulated representation of the SRETP

Week 1							
3 min	2 min	3 min	2 min	3 min	2 min	3 min	2 min
80%AT	50% Δ	80%AT	50% Δ	80%AT	50% Δ	80%AT	50% Δ

Week 2-8											
3 min	2 min	3 min	2 min	3 min	2 min	3 min	2 min	3 min	2 min	3 min	2 min
80%AT	50% Δ	80%AT	50% Δ	80%AT	50% Δ	80%AT	50% Δ	80%AT	50% Δ	80%AT	50% Δ

Supplementary Section S2.2. Tabulated representation of the structured responsive exercise training programme (SRETP) for Week 1 and Weeks 2-8**. 80%AT** Work rate (Watts) equal to 80% of work-rate at anaerobic threshold (AT). **50%Δ** Work-rate (Watts) equal to midpoint between AT and peak volume of oxygen consumption ($\dot{V}O_2$peak). All sessions also included 5-minute unloaded warm-up and 5-minute cool-down.

2.3 Visual summary of the SRETP

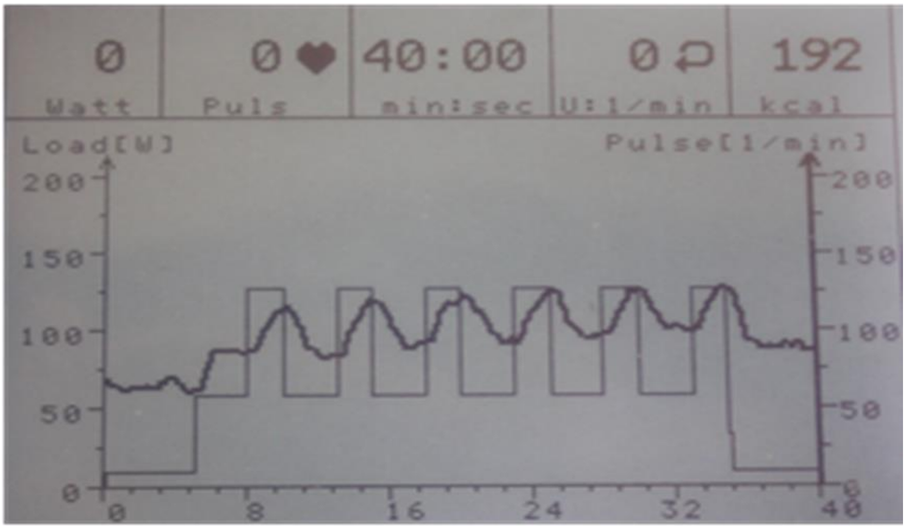

Supplementary Section S2.3 Example completed structured exercise training programme session as visible to the participant and researcher on the head of the Optibike. Work-rate (straight-line left y-axis [Watts]) with superimposed heart rate from chest monitoring of R-R interval (black line right y-axis [beats/min]), x-axis (time [minutes])

3: Biomarker analytical methodology

Following collection, EDTA plasma samples were pre-treated with the alkylating agent N-ethylmaleimide to prevent artefactual oxidation,(10) immediately centrifuged, then snap frozen in liquid nitrogen before storage at -80°C until analysis. Serum samples were allowed to stand for 30 minutes at room temperature before centrifugation, snap freezing and storage at -80°C.

Thiol redox metabolome

Total free thiols, which mostly reflect the availability of a single free cysteine group (Cys-34) of albumin, were measured as a marker of whole-body redox status (with higher values representing increased antioxidant capacity and/or enhanced propensity for disulfide reduction) and were determined spectrophotometrically using Ellman's reagent (5,5'-dithio-bis-2-nitrobenzoic acid; DTNB) and normalised to protein concentration.(11)

Free aminothiols and hydrogen sulfide (H_2S), which included the reduced and oxidised forms of glutathione (reduced glutathione [free GSH] and glutathione disulfide [GSSG]), cysteine (reduced cysteine [free Cys] and cystine [CySS]), homocysteine (reduced homocysteine [free HCys] and homocystine [HCySS]), and free and oxidised sulfide, were quantified from EDTA plasma pretreated with N-ethylmaleimide (NEM) using ultrahigh-pressure liquid chromatography-electrospray ionization-tandem mass spectrometry (UPLC-ESI-MS/MS); free and bound thiols were quantified before and after reduction of plasma aliquots by dithiothreitol as previously described(12). Redox couples were calculated e.g., free GSH/GSSG, free CyS/CySS, free HCyS/HCySS as markers of extracellular redox potential.

Lipid peroxidation

Whole-body lipid peroxidation status was measured as thiobarbituric acid reactive substances (TBARS) in serum by the malondialdehyde (MDA) colorimetric assay.(13, 14) 4-Hydroxynonenal (4-HNE) concentrations were quantified as a downstream marker of lipid peroxidation from EDTA plasma samples using the enzyme-linked immunosorbent assay (ELISA) technique (4-HNE micro-ELISA plate - Elabscience® USA [Detection range 0.63 - 40 ng/ml]).

FRAP assay

The ferric reducing ability of plasma (FRAP) was quantified as a measure of total antioxidant capacity. The FRAP assay measures the reduction of ferric (Fe^{3+}) to ferrous (Fe^{2+}) ions by the formation of an intensely blue coloured ferrous-tripyridyltriazine. Due to the acidic conditions of the FRAP assay it does not include the reducing capacity of thiols.(15) A standard curve of known concentrations of ferrous ions is used to compare absorbances of Fe^{3+} reacted plasma at 593 nm.

Nitric oxide (NO) production and metabolism biomarkers

Nitrite and nitrate concentrations were quantified from EDTA plasma following methanol precipitation of protein using a dedicated high-performance liquid chromatography system (ENO-20, Amuza Inc. USA).(16) Total

nitrosation products (RXNO) were quantified from EDTA plasma samples using gas-phase chemiluminescence detection (CLD 77am sp, EcoPhysics, Duernten, Switzerland) of bound NO, following removal of nitrite with acidified sulfanilamide and reduction of nitroso species by acidic triiodide.(17) Due to the sensitivity of the assay and the ubiquitous presences of environmental nitrite containing compounds the RXNO assay is particularly sensitive to contamination during sample processing/storage. RXNO concentrations of >100 nM are supraphysiological and were deemed extreme outliers and excluded from the final analysis.(17) Cyclic guanosine monophosphate (cGMP) was measured as a downstream marker of NO signalling. cGMP concentrations were quantified in EDTA plasma by ELISA (cGMP parameter assay kit R&D Systems).

4: Expressing the oxygen cost of exercise

During CPET the oxygen cost of performing work (exercise) can be expressed by the slope of the relationship between the oxygen uptake and work-rate ($\dot{V}O_2$/WR slope). In healthy subjects the $\dot{V}O_2$/WR (up to maximal exercise intensities) during incremental CPET is surprisingly constant with a normal range of 10.2 ±1 ml.kg^{-1}.W^{-1}.(18) A reduced $\dot{V}O_2$/WR is indicative of impaired oxygen delivery to the muscle. However although a lower $\dot{V}O_2$/WR slope represents less oxygen utilisation per Watt (suggestive of increased efficiency) it is important to consider in patients with cardiopulmonary disease, particularly at heavy/vigorous exercise intensities above the anaerobic threshold (AT), this may represent a developing oxygen debt that is then repaid in recovery.(19) In the present study recovery data was not available as, due to their lung disease, patients had significant breathlessness at peak exercise which prevented them being able to tolerate the CPET mask during recovery. In order to mitigate the impact of a developing oxygen debt during heavy/vigorous exercise intensities the oxygen cost of exercise was estimated by expressing the $\dot{V}O_2$ work-rate relationship up to the AT.

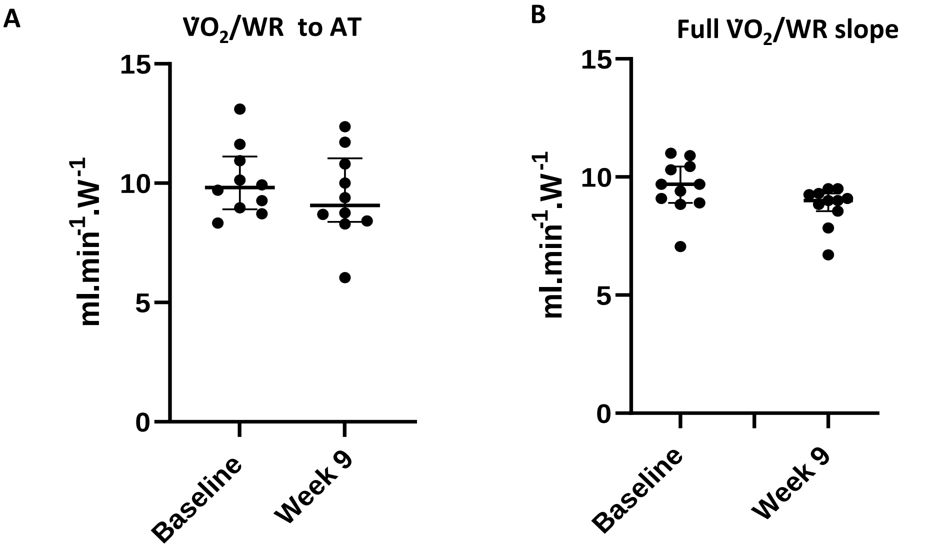

	Baseline	Week 9	p
**V̇O2/WR to the AT**	**9.93 (8.8-11.3)**	**9.07 (8.4-11.04)**	**0.515**
**Full V̇O2/WR slope**	**9.70 (8.9-10.5)**	**9.00 (8.6-9.3)**	**0.075**

Supplementary Section S4: Expressing the oxygen cost of exercise A) $\dot{V}O_2$/WR to the anaerobic threshold B) Full $\dot{V}O_2$/WR slope. Table values present median and interquartile range.

Supplementary Tables

Supplementary Table S1

Absolute contraindications	Relative contraindications
Acute myocardial infarction (3–5 days)	Untreated left main stem coronary stenosis
Unstable angina	Asymptomatic severe aortic stenosis
Uncontrolled arrhythmia causing symptoms or haemodynamic compromise	Severe untreated arterial hypertension at rest (>200 mm Hg systolic, >120 mm Hg diastolic)
Syncope	Tachyarrhythmias or bradyarrhythmias
Active endocarditis	Hypertrophic cardiomyopathy
Acute myocarditis or pericarditis	Significant pulmonary hypertension
Symptomatic severe aortic stenosis	Thrombosis of the lower extremity until treated for a minimum of 2 weeks
Suspected dissecting or leaking aortic aneurysm	Abdominal aortic aneurysm >8.0 cm
Uncontrolled asthma	Electrolyte abnormalities
Arterial desaturation at rest on room air <85%	Advanced or complicated pregnancy
Uncontrolled heart failure	Within 2 weeks of acute symptomatic pulmonary embolus

Supplementary Table S1: Absolute and Relative contradictions to Cardiopulmonary exercise testing from Levett et al. 2018 (1)

Supplementary Table S2

Control Participant Elective Surgery type	Participants (n)
Abdominal Aortic Aneurysm Repair	4
Non-malignant lower gastro-intestinal	3
Diaphragmatic hernia repair	1
Bilateral total knee replacement	1
Deep brain stimulator insertion (for Parkinson's Disease)	1

Supplementary Table S2. Tabular illustration of the indications for referral for standard care perioperative CPET of participants in the Control group.

Supplementary Table S3

	IPF n=15<br>median (IQR), % (n)	Control n=10<br>median (IQR) % (n)	p value
**Demographics**			
Age (years)	72.5 (69-80)	70.4 (62-75)	0.144
Sex (%Male)	87% (13)	80% (8)	1.000
BMI ($kg.m^{-2}$)	28.0 (26-32)	30.1 (24-35)	0.461
FEV1% predicted	84 (82-90)	91 (61-99)	0.461
FVC% predicted	78 (73-88)	92(79-0.81)	0.062
FEV1/FVC	0.82 (0.79-0.84)	0.74 (0.69-0.81)	0.008**
SpO_2 at rest	99 (97-99)	99 (97-99)	0.215
Ex-smoker	67% (n=10)	70% (n=7)	0.861
COPD	20% (3)	20% (2)	1.000
T2DM	20% (3)	10% (1)	0.504
IHD	13% (2)	50% (5)	0.075
**CPET Variables**			
AT ($ml.kg^{-1}.min^{-1}$)†	8.05 (7.4-9.9)	9.10 (8.1-11.4)	0.259
AT/$\dot{V}O_2$peak pred. (%)†	41.18 (37.1-44.3)	46.97 (41.4-51.0)	0.166
$\dot{V}O_2$peak ($ml.kg^{-1}.min^{-1}$)	11.95 (11.5-14.2)	14.17 (13.2-19.8)	0.160
$\dot{V}O_2$peak %predicted	66.7 (57.1-75.9)	64.93 (62.3-83.6)	0.807
$\dot{V}_E/\dot{V}CO_2$ slope	41.00 (35.5-55.0)	32.83 (31.1-35.8)	0.002**
$\dot{V}O_2$/WR slope	9.70 (9.0-10.3)	8.70 (7.6-9.5)	0.041*
SpO_2 at $\dot{V}O_2$peak	93 (89-96)	99 (98-99)	0.002**
Breathing Reserve (%)	38.5 (20-56)	37.0 (18-67)	0.892
Peak $\dot{V}_E$ ($L.min^{-1}$)	62.0 (48-83)	50.0 (40-81)	0.428

Supplementary Table S3: Comparison between demographics of IPF participants at baseline and Control Participants. Values represented as median (interquartile range). BMI- body mass index, FEV1- forced expiratory volume in 1 s, FVC- forced vital capacity. SpO_2-peripheral oxygen saturations by pulse oximetry, COPD- chronic obstructive pulmonary disease, T2DM- type 2 diabetes mellitus, IHD- ischaemic heart disease, AT- anaerobic threshold, $\dot{V}O_2$peak- peak volume of oxygen consumption. $\dot{V}O_2$peak pred.- $\dot{V}O_2$peak- expressed as percentage predicted, $\dot{V}_E/\dot{V}CO_2$ slope- relationship of minute ventilation ($\dot{V}_E$) to volume of carbon dioxide production ($\dot{V}CO_2$), WR- work rate. Peak $\dot{V}_E$- peak minute ventilation. *p<0.05, **p<0.01 †IPF AT n=14

Supplementary Table S4

Analyte	IPF			Control			IPF vs. Control	
	PRE (n=15)	POST (n=14)	p	PRE (n=10)	POST (n=10)	p	PRE p	POST p
**GSH/GSSG† (ratio)**	736.1 (289-1246)	456.8 (354-1077)	0.600	1855.8 (893-2454)	1337.2 (647-2345)	0.066	0.046*	0.051
**Total GSH (µM)**	3.820 (1.31-5.12)	3.655 (2.24-4.69)	0.382	2.395 (1.81-3.97)	3.245 (2.02-4.53)	0.007**	0.338	0.796
**TFT (µM)**	56.70 (40.1-69.1)	55.15 (46.2-61.1)	0.432	50.00 (37.3-62.0)	47.10 (36.1-55.5)	0.123	0.531	0.321
**TFT/Protein (µmol/g protein)**	0.880 (0.67-1.16)	0.830 (0.76-1.01)	0.141	0.785 (0.59-0.99)	0.745 (0.52-0.94)	0.740	0.261	0.192
**TBARS (µM)**	4.13 (3.49-6.61)	4.95 (3.92-7.80)	0.432	4.05 (2.90-4.84)	4.78 (3.55-6.28)	0.123	0.397	0.585
**4-HNE (ng/ml)**	8.30 (5.50-13.00)	8.15 (6.00-9.78)	0.209	7.20 (5.83-11.72)	9.60 (6.08-1.25)	0.575	0.849	0.437
**Free GSH (nM)**	3102.0 (2488-5595)	4317.0 (2427-6332)	0.510	4337.5 (2444-8716)	5424.0 (2784-9340)	0.114	0.531	0.341
**GSSG (nM)**	5.400 (3.30-10.40)	6.650 (2.20-14.48)	0.975	5.300 (0.95-7.67)	5.750 (1.18-12.48)	0.051	0.643	0.752
**Total CyS (µM)**	232.0 (197.0-260.0)	222.0 (183.8-250.5)	0.530	254.0 (217.3-286.8)	268.5 (220.8-287.5)	0.407	0.338	0.074
**Free Cys (nM)**	9486.0 (7527-10908)	7426.0 (5322-9891)	0.100	9001.0 (7305-11217)	9185.0 (6846-11218)	0.646	0.935	0.311
**CySS (nM)**	51099.0 (43229-64214)	50066.5 (41728-56889)	0.433	62530.5 (46465-74334)	52496.5 (46261-74426)	0.445	0.238	0.285
**Cys/CySS Ratio**	0.170 (0.15-0.21)	0.155 (0.12-0.21)	0.865	0.150 (0.12-0.22)	0.150 (0.12-0.21)	0.134	0.367	0.841
**Total HCyS (µM)**	10.60 (8.75-13.70)	10.19 (8.57-11.63)	0.140	14.30 (8.5-15.8)	14.55 (8.91-15.80)	0.114	0.367	0.096
**Free HCyS (nM)**	132.0 (109.0-160.0)	115.5 (95.6-142.8)	0.041*	140.5 (127.0-186.0)	142.0 (114-161)	0.241	0.567	0.312
**HCySS (nM)**	17.20 (9.7-23.3)	14.95 (5.8-22.8)	0.300	23.75 (8.6-48.8)	22.20 (6.52-48.0)	0.445	0.367	0.212
**HCys/HCySS Ratio**	8.090 (5.50-19.85	9.005 (4.47-20.69)	0.975	6.090 (3.79-13.53)	5.30 (3.18-17.22)	0.878	0.311	0.341
**Total Sulfide (µM)**	6.910 (5.68-8.20)	7.005 (5.69-10.67)	0.397	6.565 (5.58-8.81)	7.965 (5.69-8.83)	0.575	0.978	0.796
**Free Sulfide (nM)**	2574.0 (1295-3739)	2242.0 (1756-3716)	0.638	2923.0 (1213-3370)	2189.5 (1438-3184)	0.799	0.892	0.841
**Persulfide (µM)**	3.81 (3.11-5.91)	4.92 (3.62-6.14)	0.158	3.71 (3.58-6.56)	4.54 (3.64-6.27)	0.959	0.397	0.931
**FRAP (µM)**	1362.0 (1237-1502)	1344.5 (1241-1505)	0.470	1446.5 (1324-1567)	1436.0 (1137-1629)	0.074	0.216	0.508

Supplementary Table S4: Baseline pre and post cardiopulmonary exercise test (CPET) redox biomarker concentrations in IPF vs. Control participants. Results presented as median (Interquartile range). GSH/GSSG- reduced glutathione/glutathione disulfide**,** Total GSH- total glutathione (reduced + oxidised/bound GSH), TFT- total free thiols, TFT/Protein- total free thiols corrected for protein concentration, TBARS- Thiobarbituric acid reactive substances, 4-HNE- 4-Hydroxynonenal, Free GSH- reduced glutathione, GSSG- glutathione disulfide. Total Cys- Total Cysteine (reduced +oxidised/bound Cys), Free Cys- cysteine, CySS- oxidised cysteine, Total HCys- total homocysteine (reduced + oxidised/bound HCyS) homocysteine, Free HCys- homocysteine, HCySS- oxidised homocysteine, Total Sulfide- total sulfide (reduced + oxidised/bound sulfide), Free Sulfide- (reduced sulfide), Persulfide (Total – Free Sulfide), FRAP- ferric reducing ability of plasma, †GSH/GSSG Control PRE and POST n=9; IPF PRE n=14 and IPF POST n=13. *p<0.05

Supplementary Table S5

	IPF			Control			IPF vs. Control	
**Analyte**	**PRE (n=15)**	**POST (n=14)**	**p**	**PRE (n=10)**	**POST (n=10)**	**p**	**PRE p**	**POST p**
**Nitrite (µM)**	1.220 (0.43-1.37)	0.975 (0.60-1.13)	0.133	0.835 (0.70-0.98)	0.990 (0.84-1.17)	0.155	0.261	0.752
**Nitrate (µM)**	27.80 (22.4-47.0)	32.10 (26.9-46.0)	0.600	31.35 (25.8-39.9)	31.65 (25.8-48.2)	0.285	0.567	0.796
**RXNO† (nM)**	14.05 (9.37-20.2)	7.60 (3.9-15.7)	0.182	20.60 (11.45-32.05)	15.40 (6.55-29.40)	0.515	0.212	0.800
**cGMP (pmol/ml)**	179.00 (137.0-209.0)	199.50 (101.3-232.5)	0.975	151.50 (100.0-180.3)	115.00 (97.4-167.3)	0.086	0.238	0.064

Supplementary Table S5: Baseline pre and post cardiopulmonary exercise test (CPET) nitric oxide (NO) production and metabolism biomarker concentrations in IPF vs. Control participants. Results presented as median (Interquartile range). Nitrite (NO_2^-), Nitrate (NO_3^-), RXNO- total nitroso species, cGMP cyclic guanosine monophosphate. †RXNO: Control POST n=9, IPF PRE n=14, IPF POST n=13.

Supplementary Table S6

Supplementary Table S6A

**Baseline NOx**	**SpO_2 at Peak Exercise**		**Comparison**
	**<92% (n=6)**	**≥92% (n=9)**	**p**
**Nitrite PRE (µM)**	1.06 (0.65-1.57)	1.26 (0.39-1.35)	0.96
**Nitrite POST (µM)†**	0.86 (0.56-1.43)	1.03 (0.48-1.11)	0.85
**Delta Nitrite (Pre – Post)†**	-0.11 (-0.43 to +0.07)	-0.14 (-0.27 to +0.06)	0.95
**Nitrate PRE (µM)**	28.85 (19.97-71.30)	27.80 (22.9-44.5)	0.95
**Nitrate POST (µM)†**	30.25 (23.9-64.3)	36.80 (27.3-45.0)	1.00
**Delta Nitrate (Pre – Post)†**	-0.23 (-7.7 to +4.1)	1.60 (-3.61 to +5.31)	0.76

Supplementary Table S6B

**IPF (n=14)**	**Delta Nitrite (Pre – Post)**	**Delta Nitrate (Pre – Post)**
**Delta S_pO_2 Baseline (Peak Exercise - Rest)**	r = 0.09 p=0.77	r = -0.23 p=0.43

Supplementary Table S6: Comparison of nitrite and nitrate concentrations in IPF participants at baseline by peripheral oxygen saturations (S_pO_2) at peak exercise. S6A) Comparison of nitrite and nitrate concentrations stratified by S_pO_2 <92% (n=6) or ≥92% (n=9). PRE = Pre cardiopulmonary exercise test (CPET), Post = Post CPET, Delta = Pre CPET – Post CPET. †S_pO_2 ≥92% Post CPET and delta n=8. Results presented as median (interquartile range). S6B). Comparison of delta nitrite and nitrate concentrations and delta S_pO_2. Delta Nitrite/Nitrate = Pre CPET – Post CPET concentration, delta S_pO_2 = S_pO_2 at peak exercise - S_pO_2 at rest. Comparison made using Spearman's Rank co-efficient (r).

Supplementary Table S7

	Baseline	Week 9	Comparison
**SGRQ-IPF Domain**	**n=11**	**n=11**	**p value**
Symptoms	73.80 (44.8-84.2)	63.40 (33.5-75.5)	0.176
Activity	78.30 (48.1-100.0)	67.30 (48.1-88.0)	0.594
Impacts	31.30 (3.0-45.4)	31.49 (6.0-42.5)	0.678
Total Score	52.40 (23.7-64.1)	52.11 (21.7-58.7)	0.790

Supplementary Table S7. **Effect of the structured responsive exercise training programme (SRETP) on health-related quality of life score assessed using the St. George's Respiratory Questionnaire -IPF (SGRQ-IPF).** Results presented as median (interquartile range).

Supplementary Table S8

Analyte	Baseline		Week 9			Baseline vs. Week 9	
	PRE (n=11)	POST (n=10)	PRE (n=11)	POST (n=11)	p	PRE p	POST p
**GSH/GSSG† (ratio)**	516.1 (227-1246)	439.5 (288-787)	495.4 (300-1836)	480.3 (147-1063)	0.086	0.441	0.575
**Total GSH (µM)**	3.960 (1.26-5.12)	3.275 (1.38-4.61)	3.350 (1.53-4.73)	4.500 (1.96-6.09)	0.021*	0.790	0.028*
**Total Free Thiols (µM)**	52.60 (40.1-61.7)	53.40 (46.3-56.7)	63.40 (48.4-78.1)	61.20 (53.3-80.4)	0.155	0.093	0.028*
**TFT/Protein (µmol/g protein)**	0.870 (0.67-1.02)	0.795 (0.76-0.95)	1.030 (0.90-1.18)	1.050 (0.97-1.18)	0.168	0.182	0.005**
**TBARS (µM)**	4.330 (3.49-6.61)	5.475 (4.17-7.93)	5.990 (3.76-8.44)	4.410 (3.84-7.08)	0.016*	0.213	0.114
**4-HNE (ng/ml)**	8.200 (5.50-13.00)	8.150 (5.68-9.78)	7.100 (5.70-10.40)	7.700 (6.10-13.60)	0.131	0.657	0.507
**Free GSH† (nM)**	2836.0 (2056-5595)	3127.5 (1709-5710)	3058.0 (1271-6040)	3058.0 (316-6040)	0.727	0.328	0.333
**GSSG† (nM)**	4.500 (3.30-17.80)	6.650 (2.18-15.18)	5.500 (0.60-15.70)	9.600 (4.40-17.40)	0.110	0.508	0.066
**Total CyS (µM)**	250.0 (218-282)	236.5 (183-261)	244.0 (184-280)	257.0 (196-311)	0.424	0.929	0.139
**Free Cys (nM)**	9945.0 (8130-11268)	7820.0 (4427-10377)	7278.0 (5824-15641)	6403.0 (5055-13382)	0.534	1.000	0.722
**CySS (nM)**	62509.0 (50220-71077)	52260.0 (42283-63615)	59608.0 (38371-63323)	47575.0 (36041-56271)	0.213	0.790	0.508
**CyS/CySS Ratio**	0.180 (0.15-0.21)	0.145 (0.12-0.22)	0.170 (0.10-0.26)	0.190 (0.12-0.21)	0.905	0.441	0.777
**Total HcyS (µM)**	11.20 (8.95-15.90)	10.70 (9.01-12.98)	10.40 (6.73-12.30)	11.20 (9.21-12.40)	0.505	0.168	0.646
**Free HcyS (nM)**	135.0 (119-160)	110.5 (95-178)	107.0 (92-186)	102.0 (87-125)	0.878	0.248	0.648
**HCySS (nM)**	18.00 (13.4-36.9)	19.20 (9.6-27.3)	14.40 (10.5-23.6)	17.80 (10.6-26.0)	0.594	0.182	0.285
**HCys/HCySS Ratio**	6.200 (3.72-8.85)	7.100 (3.87-12.13)	4.750 (3.89-22.20)	5.800 (4.35-18.06)	0.086	0.657	0.203
**Total Sulfide (µM)**	6.150 (5.35-8.15)	7.005 (5.25-10.68)	7.390 (5.53-10.20)	7.700 (5.89-10.50)	0.859	0.182	0.541
**Free Sulfide (nM)**	2574.0 (1334-3739)	2463.5 (1808-3893)	2564.0 (1599-4562)	2358.0 (1379-4590)	0.657	0.657	0.721
**Persulfide (µM)**	3.45 (2.70-4.90)	4.67 (3.29-5.22)	5.50 (3.93-6.81)	5.02 (2.50-6.92)	0.594	0.075	0.721
**FRAP (µM)**	1290 (1237-1502)	1326 (1241-1505)	1361 (1176-1481)	1402 (1236-1525)	0.083	0.790	0.241

Supplementary Table S8: Redox biomarker concentrations at Baseline and Week 9 pre and post cardiopulmonary exercise test (CPET) for IPF patients completing the structured responsive exercise training programme (SRETP). Results presented as median (Interquartile range). GSH/GSSG- reduced glutathione/ glutathione disulfide**,** Total GSH- total glutathione (reduced + oxidised/bound GSH), TFT- total free thiols, TFT/Protein- total free thiols corrected for protein concentration, TBARS-Thiobarbituric acid reactive substances, Free GSH- reduced glutathione, GSSG- glutathione disulfide. Total Cys- total cysteine (reduced +oxidised/bound CyS), Free Cys- Reduced cysteine, CySS- oxidised cysteine, Total HCys- total homocysteine (reduced + oxidised/bound HCyS), Free HCys- reduced homocysteine, HCySS- oxidised homocysteine, Total Sulfide- total sulfide (reduced + oxidised/bound sulfide), Free Sulfide- reduced sulfide, Persulfide (Total – Free Sulfide), FRAP- ferric reducing ability of plasma. †GSH/GSSH Week 0 PRE n=10 and POST n=9, Week 9 PRE and Week 9 POST n=9. *p<0.05, **p<0.01.

Supplementary Table S9

	Baseline		Week 9			Baseline vs. Week 9	
**Analyte**	**PRE (n=11)**	**POST (n=10)**	**PRE (n=11)**	**POST (n=11)**	**p**	**PRE p**	**POST p**
**Nitrite (µM)**	1.220 (0.80-1.37)	0.975 (0.81-1.22)	1.160 (0.37-1.33)	0.660 (0.28-1.03)	0.013*	0.534	0.037*
**Nitrate (µM)**	27.70 (21.6-29.9)	30.25 (26.9-42.0)	27.70 (21.5-35.4)	27.50 (25.4-42.4)	0.286	0.799	0.878
**RXNO† (nM)**	12.30 (7.57-22.40)	6.00 (3.40-14.00)	12.95 (8.88-15.07)	12.55 (9.15-29.40)	0.374	0.594	0.263
**cGMP (pmol/ml)**	172.0 (111-209)	189.5 (98.1-241)	187.0 (118-202)	184.0 (151.0-204)	0.374	0.477	0.646

Supplementary Table S9: Nitric oxide (NO) metabolite biomarker concentrations at Baseline and Week 9 pre and post cardiopulmonary exercise test (CPET) for IPF patients completing the structured responsive exercise training programme (SRETP). Results presented as median (Interquartile range). Nitrite (NO_2^-), Nitrate (NO_3^-), RXNO- total nitroso species, cGMP cyclic guanosine monophosphate. †RXNO: Baseline PRE n=10, POST n=9. Week 4 PRE n=6, POST n=7. Week 9 PRE n=10 POST n=10. *p<0.05

Supplementary Table S10

Supplementary Table S10A

**Week 9 NOx**	**SpO_2 at Peak Exercise**		**Comparison**
	**<92% (n=5)**	**≥92% (n=6)**	**p**
**Nitrite PRE (µM)**	1.22 (0.34-1.38)	0.91 (0.54-1.67)	1.00
**Nitrite POST (µM)**	0.39 (0.27-1.14)	0.67 (0.49-0.93)	0.66
**Delta Nitrite (Pre – Post)**	-0.21 (-0.62 to -0.01)	-0.11 (-0.83 to -0.02)	0.93
**Nitrate PRE (µM)**	27.30 (18.5-39.4)	28.00 (24.3-37.9)	0.95
**Nitrate POST (µM)**	33.80 (21.6-45.3)	27.00 (22.7-39.2)	1.00
**Delta Nitrate (Pre – Post)**	5.00 (0.23-9.67)	-0.83 (-1.94 to +1.66)	0.05

Supplementary Table S10B

**IPF (n=11)**	**Delta Nitrite**	**Delta Nitrate**
**Delta S_pO_2 Week 9 (Peak Exercise - Rest)**	r = -0.08 p=0.82	r = -0.64 p=0.048*

Supplementary Table S9: Comparison of nitrite and nitrate concentrations in IPF participants at baseline by peripheral oxygen saturations (S_pO_2) at peak exercise. S10A) Comparison of nitrite and nitrate concentrations stratified by S_pO_2 <92% (n=5) or ≥92% (n=6). PRE = Pre cardiopulmonary exercise test (CPET), Post = Post CPET, Delta = Pre CPET – Post CPET. Results presented as median and interquartile range (IQR). S10B). Comparison of delta nitrite and nitrate concentrations and delta S_pO_2. Delta Nitrite/Nitrate = Pre CPET - Post CPET concentration, delta S_pO_2 = S_pO_2 at peak exercise - S_pO_2 at rest. Comparison made using Spearman's Rank co-efficient (r) *p<0.05.

Supplementary Figures

Supplementary Figure S1

IPF participant study schedule

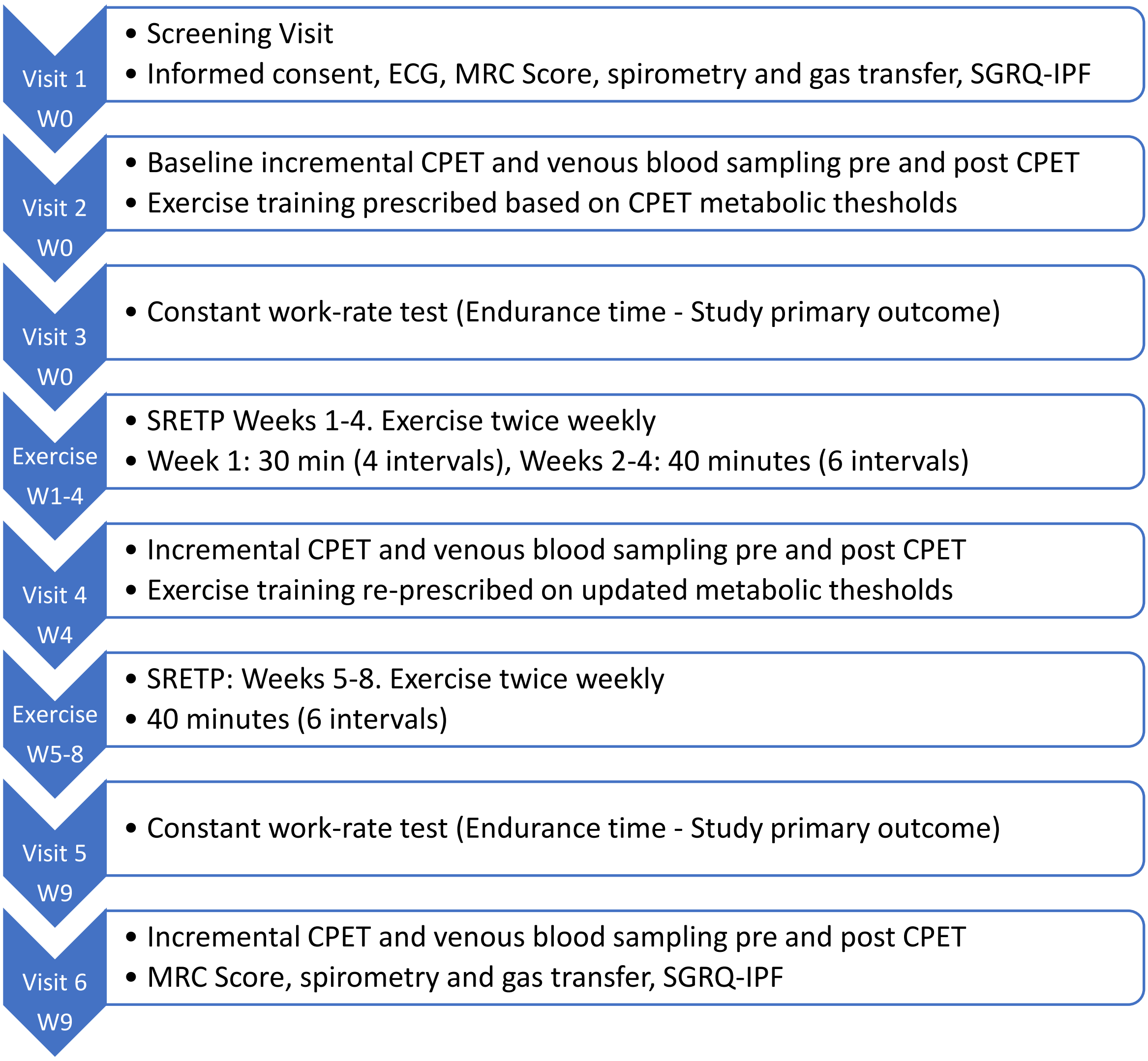

Supplementary Figure S1B:

Control participant study schedule

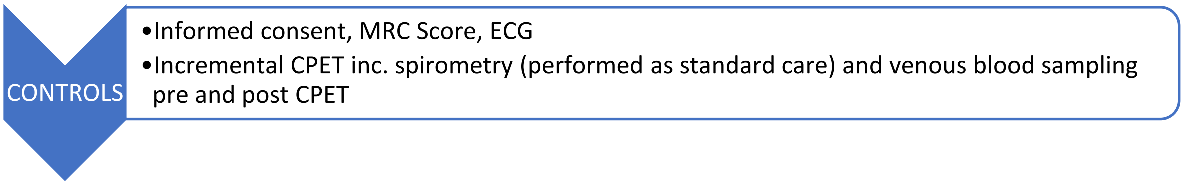

Supplementary Figure S1: Study Visit Schedule Flow diagram for IPF participants (Figure S1A) and Controls (Figure S1B). W – Week, ECG- Electrocardiogram, CPET- cardiopulmonary exercise test, MRC-Score- Medical Research Council Breathlessness Score, SGRQ-IPF- St. George's Respiratory Questionnaire IPF, SRETP: Structured responsive exercise training programme (for SRETP description see **Supplementary Section S2**).

Supplementary Figure S2

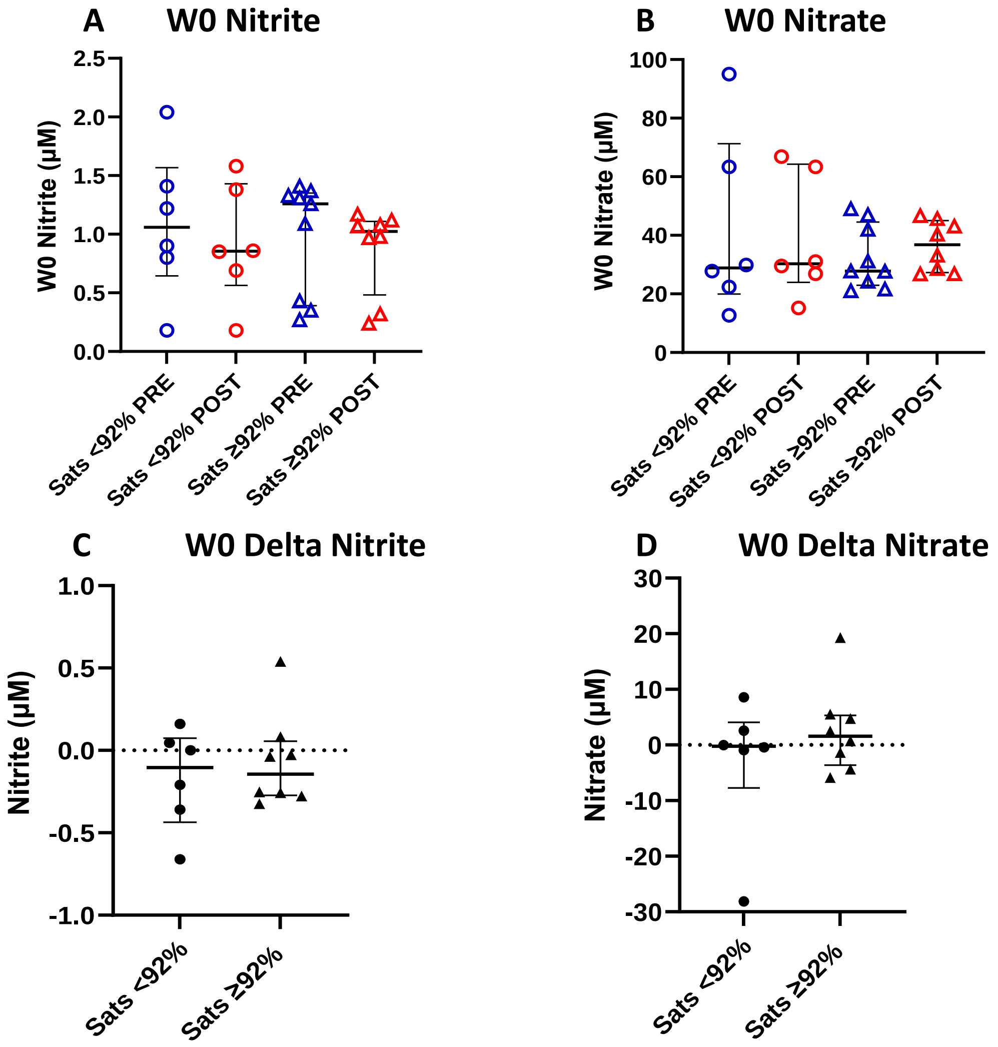

Supplementary Figure S2 Comparison of nitrite and nitrate concentrations in IPF participants at Week 0 (W0, baseline) stratified by peripheral oxygen saturations (S_pO_2) at peak exercise. Peak exercise S_pO_2 <92% vs. ≥92%. A) Nitrite concentrations B) Nitrate concentrations C) Delta (Pre CPET – Post CPET) nitrite concentrations, D) Delta nitrate concentrations. S_pO_2<92% (circles) n=6 vs. S_pO_2≥92% (triangles) n=9 (n=8 for S_pO_2≥92% Post CPET and delta). PRE (blue circles/triangles) = Pre cardiopulmonary exercise test (CPET), Post (red circles/triangles) = Post CPET.

Supplementary Figure S3

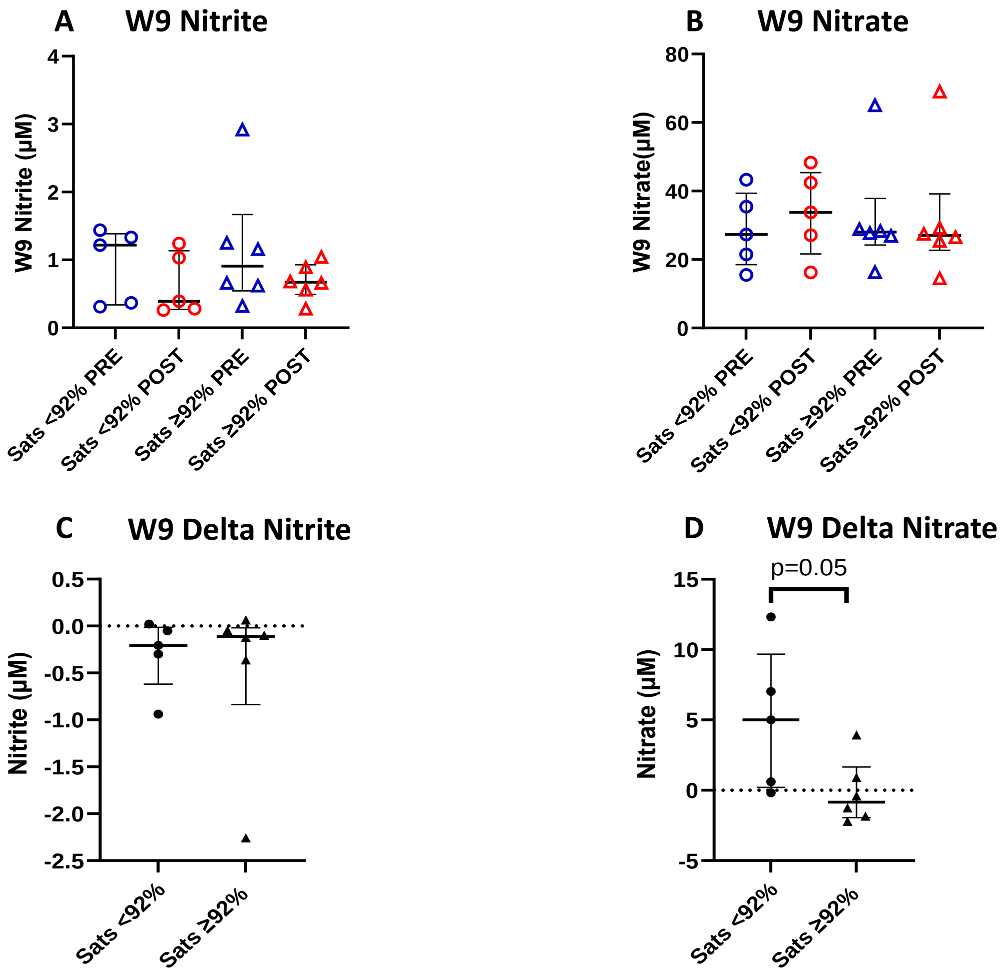

Supplementary Figure S3 Comparison of nitrite and nitrate concentrations in IPF participants at Week 9 (W9) stratified by peripheral oxygen saturations (S_pO_2) at peak exercise. Peak exercise S_pO_2: <92% vs. ≥92% A) Nitrite concentrations, B) Nitrate concentrations C) Delta (Pre CPET – Post CPET) nitrite concentrations, D) Delta nitrate concentrations. S_pO_2 <92% (circles) n=5 vs. ≥92% (triangles) n=6. PRE (blue circles/triangles) = Pre cardiopulmonary exercise test (CPET), Post (red circles/triangles) = Post CPET.

Supplementary References

1. Levett DZH, Jack S, Swart M, Carlisle J, Wilson J, Snowden C, et al. Perioperative cardiopulmonary exercise testing (CPET): consensus clinical guidelines on indications, organization, conduct, and physiological interpretation. Br J Anaesth. 2018;120(3):484-500.
2. American Thoracic Society, American College of Chest Physicans. ATS/ACCP Statement on cardiopulmonary exercise testing. Am J Respir Crit Care Med. 2003;167(2):211-77.
3. Wasserman K, Hansen J, Sue DY, Stringer W, Sietsema K, Sun X-G, et al. Principles of exercise testing and interpretation: Including pathophysiology and clinical applications: Fifth edition: Philadelphia: Lippincott Williams and Wilkins; 2011.
4. Quanjer PH, Stanojevic S, Cole TJ, Baur X, Hall GL, Culver BH, et al. Multi-ethnic reference values for spirometry for the 3-95-yr age range: the global lung function 2012 equations. The European respiratory journal. 2012;40(6):1324-43.
5. Graham BL, Steenbruggen I, Miller MR, Barjaktarevic IZ, Cooper BG, Hall GL, et al. Standardization of Spirometry 2019 Update. An Official American Thoracic Society and European Respiratory Society Technical Statement. American Journal of Respiratory and Critical Care Medicine. 2019;200(8):e70-e88.
6. Graham BL, Brusasco V, Burgos F, Cooper BG, Jensen R, Kendrick A, et al. 2017 ERS/ATS standards for single-breath carbon monoxide uptake in the lung. European Respiratory Journal. 2017;49(1):1600016.
7. Stenton C. The MRC breathlessness scale. Occupational Medicine. 2008;58(3):226-7.
8. Yorke J, Jones PW, Swigris JJ. Development and validity testing of an IPF-specific version of the St George's Respiratory Questionnaire. Thorax. 2010;65(10):921-6.
9. Slade SC, Dionne CE, Underwood M, Buchbinder R. Consensus on Exercise Reporting Template (CERT): Explanation and Elaboration Statement. Br J Sports Med. 2016;50(23):1428-37.
10. Ellis R. The use of N-ethylmaleimide in stabilizing and measuring inorganic sulphur compounds. Biochemical Journal. 1968;110(3):43P.
11. Koning AM, Meijers WC, Pasch A, Leuvenink HGD, Frenay AS, Dekker MM, et al. Serum free thiols in chronic heart failure. Pharmacol Res. 2016;111:452-8.
12. Sutton TR, Minnion M, Barbarino F, Koster G, Fernandez BO, Cumpstey AF, et al. A robust and versatile mass spectrometry platform for comprehensive assessment of the thiol redox metabolome. Redox Biology. 2018;16:359-80.
13. Tsikas D. Assessment of lipid peroxidation by measuring malondialdehyde (MDA) and relatives in biological samples: Analytical and biological challenges. Anal Biochem. 2017;524:13-30.
14. Aguilar Diaz De Leon J, Borges CR. Evaluation of Oxidative Stress in Biological Samples Using the Thiobarbituric Acid Reactive Substances Assay. J Vis Exp. 2020(159).
15. Benzie IF, Strain JJ. The ferric reducing ability of plasma (FRAP) as a measure of "antioxidant power": the FRAP assay. Anal Biochem. 1996;239(1):70-6.
16. Rassaf T, Bryan NS, Kelm M, Feelisch M. Concomitant presence of N-nitroso and S-nitroso proteins in human plasma. Free Radic Biol Med. 2002;33(11):1590-6.
17. Feelisch M, Rassaf T, Mnaimneh S, Singh N, Bryan NS, Jourd'Heuil D, et al. Concomitant S-, N-, and heme-nitros(yl)ation in biological tissues and fluids: implications for the fate of NO in vivo. Faseb j. 2002;16(13):1775-85.
18. Hansen JE, Sue DY, Oren A, Wasserman K. Relation of oxygen uptake to work rate in normal men and men with circulatory disorders. The American Journal of Cardiology. 1987;59(6):669-74.
19. Mitchell SH, Steele NP, Leclerc KM, Sullivan M, Levy WC. Oxygen Cost of Exercise Is Increased in Heart Failure After Accounting for Recovery Costs*. Chest. 2003;124(2):572-9.
